# Deciphering the regulatory mechanism and therapeutic potential of ECM degradation in intervertebral disc degeneration via multi-omics integration

**DOI:** 10.3389/fimmu.2026.1809762

**Published:** 2026-04-22

**Authors:** Lei Feng, Wei Zhong, Wenhua Liu, Xiang Guo, Leilei Wu

**Affiliations:** 1 Department of Joint Surgery, Affiliated Hospital of Shandong Second Medical University, Weifang, China; 2 Department of Spinal Surgery, Affiliated Hospital of Shandong Second Medical University, Weifang, China

**Keywords:** extracellular matrix degradation, immune microenvironment, inflammation, intervertebral disc degeneration, machine learning, pravastatin sodium

## Abstract

**Background:**

Intervertebral disc degeneration (IVDD) is a major cause of chronic low back pain, characterized not only by extracellular matrix (ECM) degradation but also by a chronic low-grade inflammatory response. The crosstalk between immune microenvironment dysregulation and protease-driven ECM breakdown remains poorly understood, hindering the development of targeted therapies.

**Methods:**

We integrated multiple transcriptomic datasets to map the landscape of ECM-degrading proteases in IVDD. Machine learning algorithms (LASSO, Random Forest, SVM) were employed to identify key regulatory genes. Their association with immune cell infiltration and inflammatory pathways was investigated. A diagnostic ridge regression model was constructed, and molecular docking was performed to screen for potential therapeutics. The top candidate, pravastatin sodium, was validated *in vitro* and *in vivo* for its effects on ECM preservation and inflammation modulation.

**Results:**

MMP3 and ADAMTS1 were identified as core genes driving ECM degradation, and their expression was strongly correlated with the abundance of pro-inflammatory immune cells and activation of inflammatory pathways. The model based on these genes effectively distinguished degenerated discs. Single-cell analysis revealed that high-degradation nucleus pulposus cells exhibited enhanced pro-inflammatory intercellular communication and metabolic reprogramming towards a pro-inflammatory state. Molecular docking confirmed high binding affinity of pravastatin sodium to MMP3 and ADAMTS1. Experimental validation demonstrated that pravastatin sodium alleviated IVDD by reducing ECM degradation, suppressing pro-inflammatory cytokine (e.g., IL-1β) expression, and inhibiting apoptosis.

**Conclusion:**

Our study delineates a critical network linking ECM degradation to immune microenvironment activation in IVDD, with MMP3 and ADAMTS1 serving as central hubs. Pravastatin sodium emerges as a promising therapeutic agent capable of disrupting this pathogenic network, offering a novel immunomodulatory strategy for slowing disc degeneration.

## Graphical Abstract

**Figure:**
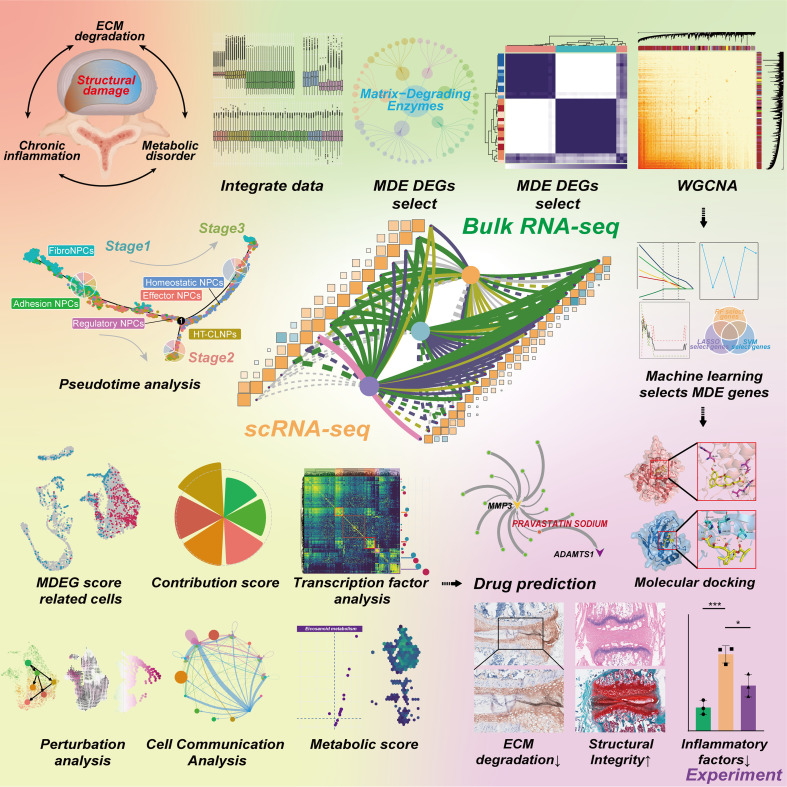

## 1 Introduction

Intervertebral disc degeneration (IVDD) represents a major contributor to chronic low back pain, one of the leading causes of disability worldwide (1). The intervertebral disc plays a crucial role in maintaining spinal flexibility and shock absorption (2). However, with aging and repetitive mechanical stress, the disc undergoes progressive degenerative changes that compromise its structural integrity and function (3, 4). This degeneration is primarily driven by the degradation of the extracellular matrix (ECM), a network of proteins that provides support and cushioning to the disc. As ECM components, such as collagen and aggrecan, break down, the IVD loses its ability to retain water and maintain its mechanical properties, leading to reduced disc height, altered biomechanics, and pain (5, 6). This disruption of the ECM not only accelerates the loss of disc function but also contributes to the inflammatory processes that further promote tissue breakdown, making IVDD a multifaceted challenge for both prevention and treatment (7, 8).

The ECM in the intervertebral disc is primarily composed of collagen II, aggrecan, and proteoglycans (9, 10). During IVDD, altered homeostasis within the disc leads to the degradation of these ECM components (11, 12). A major contributor to ECM degradation in IVDD is the increased activity of various proteolytic enzymes, including matrix metalloproteinases (MMPs), a disintegrin and metalloproteinases with thrombospondin motifs (ADAMTS), and cathepsins (5, 6, 13, 14). These enzymes are upregulated in response to pro-inflammatory cytokines and cellular stress (15–18). These conditions are often linked to the senescence and apoptosis of nucleus pulposus and annulus fibrosus cells (19, 20).

MMPs and ADAMTS proteases target collagen II and aggrecan, respectively, leading to the breakdown of the ECM’s structural integrity (13). Additionally, increased expression of these proteases results in the accumulation of bioactive fragments, which further improve ECM degradation (21, 22). This protease-driven cascade is central to the pathogenesis of IVDD and the formation of the degenerative phenotype in disc cells (7). Studies have shown that inhibiting specific proteases, such as MMPs and ADAMTS, can mitigate ECM degradation and slow the progression of IVDD (5, 23–25). These findings suggest that targeting these proteases may offer therapeutic potential in treating disc degeneration.

Although substantial research has focused on the individual roles of MMPs and ADAMTS in ECM degradation, a clear and comprehensive understanding of how these proteases interact in the context of IVDD remains lacking. Furthermore, although inflammatory signaling has been linked to the upregulation of these proteases, the precise molecular interactions and regulatory mechanisms between the different proteases and inflammatory mediators are not fully understood. Additionally, while protease inhibitors have shown promise in slowing the progression of IVDD, the identification of key molecular targets within the proteolytic pathways that drive ECM degradation is still limited.

In this study, we aim to (i) map the landscape of ECM-degrading proteases and their relationship with the immune microenvironment in IVDD using integrated transcriptomics; (ii) identify master regulatory genes that couple ECM degradation to inflammatory pathways via advanced machine learning; (iii) decode the pro-inflammatory communication and metabolic profile of degradation-active disc cells at single-cell resolution; and (iv) evaluate the therapeutic potential of targeting this integrated axis with the repurposed drug, pravastatin sodium. Through this integrated approach, we aim to provide insights into the molecular mechanisms driving IVDD and identify potential targets for future therapeutic intervention.

## 2 Materials and methods

### 2.1 Dataset acquisition

This research used six datasets sourced from the Gene Expression Omnibus (GEO). These datasets comprised mRNA expression data from GSE167199, GSE176205, GSE34095, GSE56081, and GSE70362, as well as single-cell transcriptomic data from GSE165722.

The study concentrated on a range of key protease families, including MMPs (26, 27), A Disintegrin and Metalloproteinase (ADAM) proteins, the ADAMTS family (28), serine proteases and their activators (29, 30), cathepsins (31), and glycosidases (32–36)(**Supplementary Material 1**: **Supplementary Table 1**).

### 2.2 Data processing and identification of key ECM-degrading protease genes

All statistical analyses were performed using R software. To enable integrative analysis across different platforms, batch effects resulting from the combination of microarray (GSE34095, GSE56081, GSE70362) and RNA-seq (GSE167199, GSE176205) data were eliminated using the Rank-In method (37), which is specifically designed for cross-platform integration. Principal component analysis (PCA) was conducted to visualize the removal of batch effects, utilizing the FactoMineR and factoextra R packages. Differentially expressed genes (DEGs) between the control and IVDD groups were determined using the limma package, with thresholds set at |log2(fold change)| > 0.05 and adj.P.Val < 0.05. The overlap between protease-related genes and the DEGs was assessed using the UpSetR package. The LASSO algorithm, implemented through the glmnet package, was employed to identify significant protease types associated with IVDD. Additionally, the random forest (RF) algorithm, available in the randomForest package, was used to integrate multiple decision trees in an ensemble learning approach. This approach improved model accuracy and refined the list of potential proteases. Genes that were common between the LASSO model and those with a mean Gini score reduction greater than 2 from the RF model were considered key protease types.

### 2.3 Gene set enrichment analysis

Gene set enrichment analysis (GSEA) was performed using the clusterProfiler package (version 4.2.2). Genes were ranked according to their log_2_ fold change values derived from the differential gene expression (DEG) analysis. The reference database for the analysis consisted of the Kyoto Encyclopedia of Genes and Genomes (KEGG) pathway gene sets. Enrichment was considered significant if the normalized enrichment score (NES) had an absolute value greater than 1 and the false discovery rate (FDR) q-value was less than 0.05. The results of the GSEA were visualized using the GseaVis package (version 0.0.9).

### 2.4 Unsupervised clustering

Unsupervised clustering was carried out using the ConsensusClusterPlus package with 100 iterations to ensure the stability of the results. Clustering was based on the Pearson distance metric, and PAM algorithm was applied. The number of clusters was tested within the range of 2 to 10, and the optimal number of clusters was determined by examining the consensus matrix. The consistency and stability of the clusters were further evaluated using cumulative distribution functions (CDF) and delta area plots. Principal component analysis (PCA) was used to visualize the separation of the clusters and to help determine the best K value.

### 2.5 Single-gene set variation analysis and immune infiltration analysis

Gene set variation analysis (GSVA) was performed using the GSVA package. A protease gene set (**Supplementary Material 1**: **Supplementary Table 1**) served as the reference for this analysis. The GSVA function was applied to calculate protease scores for each sample. Additionally, feature genes from single-cell data were used to quantify the abundance of 14 different cell types within the samples using the GSVA algorithm. The visualization of cell infiltration levels in the intervertebral disc tissue was done using the pheatmap package. Pearson correlation coefficients between the expression of MMP3 and ADAMTS1, Ridge scores obtained from Ridge regression, protease scores from GSVA, and the abundance of disc cell types were calculated using the ggcor package.

### 2.6 Weighted gene co-expression network analysis

WGCNA(Weighted gene co-expression network analysis) was performed using the WGCNA software package to identify gene modules and construct gene co-expression networks, facilitating the discovery of key genes within regulatory networks. Gene expression data were first ranked based on the median absolute deviation, and the top 5000 genes were selected for further analysis. The appropriate soft threshold for the network was determined to be 7 using the pickSoftThreshold function. Genes with similar expression profiles were grouped into modules through average linkage hierarchical clustering, using dissimilarity measures derived from the topological overlap matrix. The correlation between each gene module and the protease score was assessed, and the module with the highest correlation was selected for further investigation.

### 2.7 Identification of key regulator genes related to protease following IVDD using machine learning models

To identify crucial genes regulating protease activity after IVDD and to build a predictive regression model, protease-related genes with differential expression were selected using the LASSO method via the glmnet package (v4.1-3). Additionally, the support vector machine recursive feature elimination (SVM-RFE) method, implemented with the caret package, was used to iteratively remove less relevant features, optimizing the model. Random Forest (RF) analysis, performed with the randomForest package (v4.7-1), was applied to further refine potential biomarkers. Genes that appeared consistently in the LASSO, SVM-RFE, and RF models (with a Gini reduction > 2) were considered key genes.

A regression model based on the identified protease-related regulatory genes was developed using ridge regression (with alpha set to 0) in the glmnet function. The protease score was calculated using the formula: protease Ridge score = 3.79 × MMP3 + 13.98 × ADAMTS1 – 119.42. The model’s performance was validated with receiver operating characteristic curves, generated using the pROC package in the dataset.

### 2.8 Single-cell RNA sequencing data analysis

For downstream analysis of single-cell transcriptomic data from GSE165722, the Seurat package was used. Clustering and visualization in two dimensions were achieved using uniform manifold approximation and projection (UMAP) for dimensionality reduction. Differential gene expression analysis was conducted using the FindAllMarkers and MAST functions. To assess the similarity between single-cell and bulk datasets, the SCISSOR package was applied. Pearson correlation coefficients were used to measure the similarity between individual cells and batch samples. A regression model was applied to optimize the phenotype-related correlation matrix, enabling the identification of important cells with specific phenotype similarities. Finally, intercellular communication analysis was conducted and visualized using the CellChat package.

Metabolic analysis of the single-cell data was performed using the Python Compass package (v0.9.10.2) and the scMetabolism package (version 0.2.1). The RcisTarget human database was downloaded from (https://resources.aertslab.org/cistarget/) to construct the transcription factor regulatory network. This network was built using the SCENIC package (v1.3.1), and transcription factor activation was assessed using the AUCell algorithm. Regulatory submodules were identified based on metrics of connectivity specificity. The final intercellular communication analysis was carried out and visualized using the CellChat package (v1.6.1).

### 2.9 Gene selection through single-cell pseudotime analysis

Single-cell pseudotime trajectory analysis was performed to investigate dynamic changes in cell states during intervertebral disc degeneration and to identify key genes. The Monocle2 tool (version 2.22.0) was used to build the cell trajectory. Initially, a CellDataSet object was created, and genes expressed in at least 10 cells were retained after quality control. The DDRTree method was applied for dimensionality reduction, which projected the cells into a two-dimensional space and ordered them along a pseudotime axis. Differential expression analysis was conducted along this pseudotime axis using the differentialGeneTest function. Genes with a q-value (after FDR correction) less than 0.01 were identified as significantly associated with pseudotime.

### 2.10 Molecular docking analysis

Enrichment analysis was conducted using drug–gene interaction data from the Drug Signatures Database, which served as the background gene set, to identify potential drugs interacting with core genes. The two-dimensional molecular structures of selected compounds were retrieved from the PubChem database, and protein structures were obtained from UniProt. Flexible ligand docking simulations were performed using AutoDock4 software, where proteins were treated as rigid and small molecules as flexible in the PDBQT file format. The results of the molecular docking were visualized using PyMOL software.

### 2.11 Ethical statement

The animal study conducted in this research received approval from the Ethics Committee of the Affiliated Hospital of Shandong Second Medical University (Weifang, China; Approval No.: 2025SDL827). All animal experiments were performed in accordance with the “Animal Research: Reporting of *In Vivo* Experiments” (ARRIVE) guidelines.

### 2.12 Animal model

Female C57BL/6J mice, aged 8 weeks and weighing between 18 and 20 grams, were obtained from Beijing Vital River Laboratory Animal Technology Co., Ltd. (Beijing, China, License No.: SCXK(Beijing)2021-0006). The mice were housed in groups under standard conditions with ad libitum access to food and water. The housing environment maintained a 12-hour light/dark cycle, a temperature range of 20-25 °C, and relative humidity of 40-60%. The mice were randomly allocated into three experimental groups: the sham surgery group, the IVDD group, and the treatment group (n = 3 per group).

### 2.13 Mouse IVDD model

The IVDD model was created through a surgical procedure performed under sterile conditions. Mice were anesthetized using a small-animal anesthesia system. Isoflurane was delivered via a calibrated vaporizer into an induction chamber at a concentration of 1.5% (v/v) in oxygen, with a fresh gas flow rate of 2 L/min. The anesthetic state was assessed by monitoring the respiratory rate (maintained at 30–40 breaths per minute) and by confirming the absence of a pedal withdrawal reflex. Once fully anesthetized, mice were removed from the chamber, and surgery commenced.

For the surgery, the mice were placed in a prone position, and a midline longitudinal incision was made along the dorsal aspect. After the L3/4 intervertebral disc was exposed, the left facet joint between the third and fourth lumbar vertebrae was removed. A 26-gauge needle was then inserted 1.0 mm into the intervertebral disc, parallel to the endplate, and held in place for 30 seconds to induce disc degeneration (IVDD group). The muscle tissue was sutured with 3–0 silk thread, and the skin was closed using 4–0 nylon sutures.

Mice in the treatment group received intraperitoneal injections of pravastatin sodium (10 mg/kg) once daily for six weeks, while the IVDD group received saline injections according to the same schedule.

### 2.14 Histological staining

The intervertebral discs and surrounding vertebrae were harvested and processed for sectioning. The tissue sections were stained with Safranin O (Solarbio, Cat#G1371, Beijing, China) and a Modified Hematoxylin-Eosin (HE) Stain Kit (Solarbio, Cat#G1121, Beijing, China). Histological evaluation was performed using a scoring system based on five criteria: cell count in the annulus fibrosus, morphology of the annulus fibrosus (rupture or serpentine fibers), integrity of the boundary, cell count in the nucleus pulposus, and morphology of the nucleus pulposus. Each parameter was scored on a scale from 1 to 3, with the maximum possible total score being 15.

### 2.15 Isolation and culture of NPCs

Nucleus pulposus cells (NPCs) were isolated from euthanized mice. Euthanasia was performed by cervical dislocation under deep anesthesia induced by isoflurane (4% v/v in oxygen). The intervertebral discs (IVDs) were harvested using a scalpel, and the annulus fibrosus was incised to expose and retrieve the nucleus pulposus tissue. The gel-like tissue was minced and digested in 0.25% trypsin-EDTA (Solarbio, Beijing, China) for 2 hours. After digestion, the sample was passed through a 70 µm mesh to separate the cells from tissue debris. The cells were then washed with PBS, centrifuged at 135g for 3 minutes, and cultured in Dulbecco’s Modified Eagle Medium (DMEM, Gibco, Gaithersburg, USA) supplemented with 100 µg/mL streptomycin, 100 U/mL penicillin (P1400-100, Solarbio, Beijing, China), and 10% fetal bovine serum (Gibco, Gaithersburg, USA). When the cells reached 80%-90% confluence, they were passaged using 0.25% trypsin-EDTA. The culture medium was refreshed every two days. Cells used for experiments were from passages 1 to 3.

### 2.16 Immunohistochemistry

Paraffin-embedded tissue sections were dewaxed in environmental-friendly de-waxing agent (10 minutes each in three changes) and rehydrated in a gradient alcohol series (100%, 95%, 75% ethanol, 5 minutes each). After washing with TBS (3 times, 3 minutes each), antigen retrieval was performed using PH9.0 EDTA buffer in a microwave at high power for 3 minutes, repeated three times. After cooling, the sections were washed with TBS, treated with 3% H2O2 for 15 minutes at room temperature, and then incubated with 10% normal goat serum for 30 minutes at room temperature. The following primary antibodies were employed: Collagen 2 (1:1000; ArigoBio; Cat#ARG20787), Aggrecan (1:400; PTG; Cat#13880-1-AP), IL-1β (1:100; PTG; Cat#26048-1-AP).

### 2.17 TUNEL assay for NPCs apoptosis

To assess apoptosis in nucleus pulposus cells (NPCs), terminal deoxynucleotidyl transferase dUTP nick end labeling (TUNEL) assays were conducted using randomly selected nucleus pulposus samples from all experimental groups. A TUNEL apoptosis detection kit (Beyotime, China) was used to enzymatically label DNA strand breaks and evaluate apoptosis in NPCs. In brief, the nucleus pulposus sections were incubated in 100 µmol H_2_O_2_ at room temperature for 12 hours, followed by rinsing with PBS. Subsequently, 50 μL of TUNEL solution was applied, and the sections were incubated for one hour in a humidified chamber. Apoptotic NPCs were quantified by counting the TUNEL-positive cells.

### 2.18 Statistical analysis

The sample size was not predetermined using statistical methods; however, the number of samples in our study is comparable with those reported in previously published work (38). No animals or data points were excluded from the analysis, which was conducted by researchers blinded to the experimental conditions. Statistical analyses were performed using GraphPad Prism 8 (v8.0.2). For comparisons between multiple groups, a one-way analysis of variance (ANOVA) followed by Tukey’s *post hoc* test was employed. Results are expressed as mean ± standard deviation (SD), and statistical significance was defined as P-values less than 0.05. Notations used include ns (not significant); *P < 0.05; **P < 0.01; ***P < 0.001; ****P < 0.0001.

## 3 Results

### 3.1 Data integration and construction of a comprehensive protease landscape related to ECM degradation

**Figure 1:**
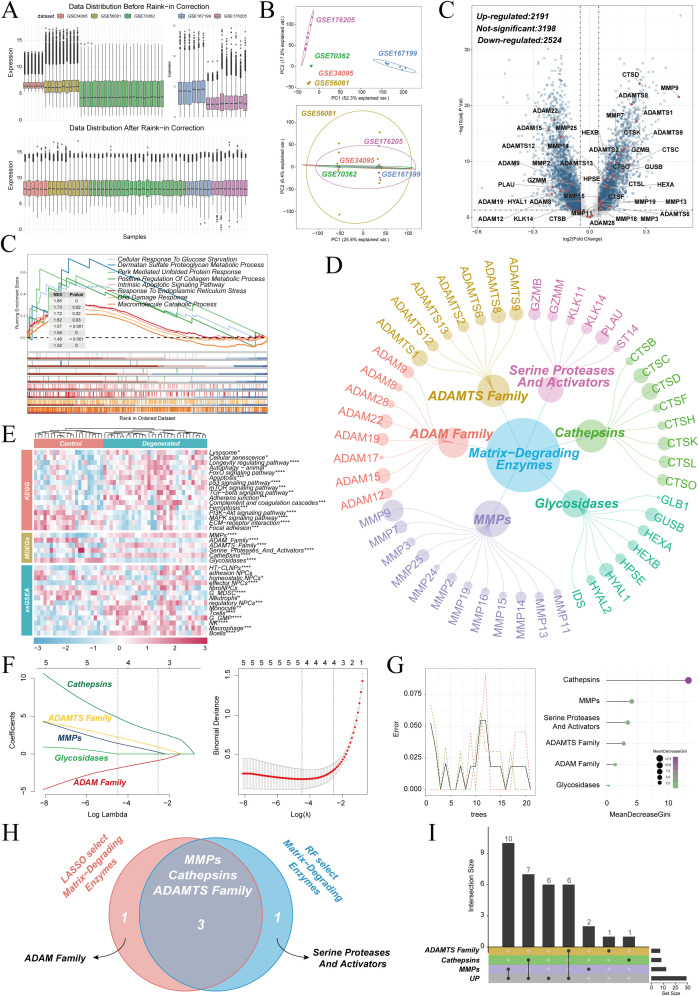
Multi-cohort identification of ECM-degrading proteases in IVDD. **(A)** Boxplots of five GEO datasets before and after Rank-in batch correction. **(B)** PCA showing improved inter-dataset consistency after correction. **(C)** Volcano plot of differentially expressed genes between degenerated and non-degenerated NP tissues (red dots: ECM-degrading proteases). **(D)** GSEA enrichment plots comparing degenerated and non-degenerated NP tissues. **(E)** Network of 49 IVDD-related protease genes grouped into six families. **(F)** GSVA heatmap of pathway, protease-family and immune/cell-type signatures in control versus degenerated group. **(G, H)** Feature selection of protease families by LASSO and random forest. **(I)** Overlap of selected families highlighting MMPs, cathepsins and ADAMTS as core proteases. **(J)** UpSet plot intersecting up-regulated DEGs with core protease genes.

Five independent transcriptomic datasets from the GEO database (GSE167199, GSE176205, GSE34095, GSE56081, GSE70362) were integrated to delineate the molecular landscape of ECM degradation during IVDD. Batch effects were corrected using the Rank-in method (**Figure 1A**). PCA was then used to confirm a high degree of consistency among the corrected datasets, thereby providing a robust basis for subsequent joint analyses (**Figure 1B**). Differential expression analysis was performed between degenerated and normal nucleus pulposus tissues. ECM degradation–related protease genes were highlighted in red in the volcano plot. A large number of protease genes were significantly upregulated in degenerated tissues, indicating widespread involvement of these enzymes in the occurrence of IVDD (**Figure 1C**).

GSEA was performed to further characterize the biological features of degenerated nucleus pulposus. The analysis showed that pathways related to the response to endoplasmic reticulum stress, the PERK-mediated unfolded protein response, and the cellular response to glucose deprivation were significantly enriched in the degenerated group. These pathways were inferred to inhibit normal protein folding in the endoplasmic reticulum and to reduce collagen synthesis, and they were also predicted to activate multiple ECM-degrading proteases, thereby aggravating metabolic imbalance of the extracellular ECM. Metabolic dysregulation of dermatan sulfate proteoglycans was particularly prominent, indicating that disruption of the proteoglycan network constitutes a key structural basis for the marked loss of intervertebral disc height (**Figure 1D**). Six classes of ECM-degrading proteases were defined on the basis of previous studies: ECM metalloproteinases (MMPs), ADAM proteases, ADAMTS proteases, serine proteases and their activators, cathepsins, and glycosidases. A set of 49 genes, termed Matrix-Degrading Enzymes genes (MDEG), that belong to these six protease families was compiled through literature curation. The genes and their classification are shown in **Figures 1E**, and the corresponding literature sources are provided in the **Supplementary Materials 1**.

GSVA-based pathway activity analysis was used to reveal deeper pathological alterations in degenerated tissues. Marked activation of the lysosomal pathway was associated with increased cathepsin-mediated degradation of collagen and proteoglycans. Cellular senescence and p53 signaling were concomitantly upregulated. Autophagy and FoxO signaling were activated as hypoxia-adaptive mechanisms and further exacerbated lysosome-dependent ECM breakdown. Abnormal mTOR signaling suppressed energy metabolism and, together with dysregulated adherens junctions and aberrant TGF-β signaling, impeded ECM synthesis and compromised structural integrity. Activation of the complement cascade established a positive feedback loop between inflammation and degradation and ultimately drove collapse of the disc ECM. These six protease classes were scored, and all of them differed significantly between degenerated and non-degenerated groups. Cell infiltration analysis showed a marked increase in multiple inflammation-related cell types and specific NPC subpopulations in the degenerated group (**Figure 1F**), consistent with these findings and underscoring the close association between the immune microenvironment and ECM degradation.

LASSO regression and random forest algorithms were applied in combination for feature selection to identify core protease among the many candidates (**Figures 1G, H**). The results indicated that MMPs, cathepsins, and ADAMTS played more critical roles in ECM degradation during IVDD (**Figure 1I**). Twenty-three core ECM degradation genes were identified by intersecting the upregulated DEGs with these three core protease genes (**Figure 1J**). These genes provide a set of targets for subsequent in-depth investigations.

### 3.2 Unsupervised clustering of IVDD subtypes based on ECM-degrading genes

**Figure 2:**
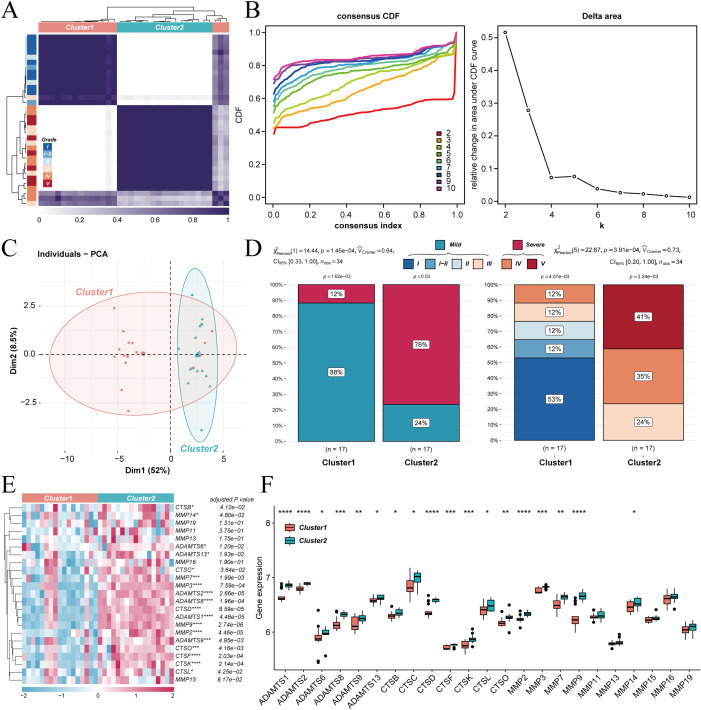
Unsupervised clustering of IVDD subtypes. **(A)** Heatmap of unsupervised clustering using the PAM algorithm. **(B)** Consensus CDF and delta area plot illustrating the optimal number of clusters. **(C)** Principal component analysis showing distinct transcriptomic profiles between the two clusters. **(D)** Bar plots indicating the relationship between clusters and IVDD severity. **(E)** Heatmap of differentially expressed ECM degradation genes between two clusters. **(F)** Boxplots showing the expression of key ECM degradation genes in the two clusters. *p < 0.05,**p < 0.01, ***p < 0.001, ****p < 0.0001.

The relationship between ECM degradation gene expression patterns and the severity of IVDD was investigated. Unsupervised clustering analysis was performed on 34 samples with defined Pfirrmann grades from the GSE56081 and GSE70362 datasets. The PAM algorithm divided all samples into two distinct subtypes, which exhibited marked differences (**Figures 2A, B**). Principal component analysis confirmed significant transcriptomic heterogeneity between the two subtypes (**Figure 2C**). Notably, the ECM degradation based molecular classification aligned with the degree of disc degeneration. Cluster 1 predominantly contained samples with mild degeneration, exhibiting Pfirrmann grades I–III, while Cluster 2 mainly consisted of samples with severe degeneration, exhibiting grades IV–V (**Figure 2D**). These findings suggest that the overall expression patterns related to ECM degradation correlate strongly with IVDD severity.

The specific genes that drove the divergence between the two subtypes were further dissected. A heatmap of differentially expressed genes was plotted, and their expression patterns were visualized using boxplots. Eighteen core genes were identified as significantly upregulated in Cluster 2 (**Figures 2E, F**). These genes form a core molecular signature that distinguishes mild from severe degeneration. They also provide a precise set of targets for further investigation into the mechanisms underlying IVDD progression.

### 3.3 WGCNA analysis of ECM degradation-related genes in IVDD

**Figure 3:**
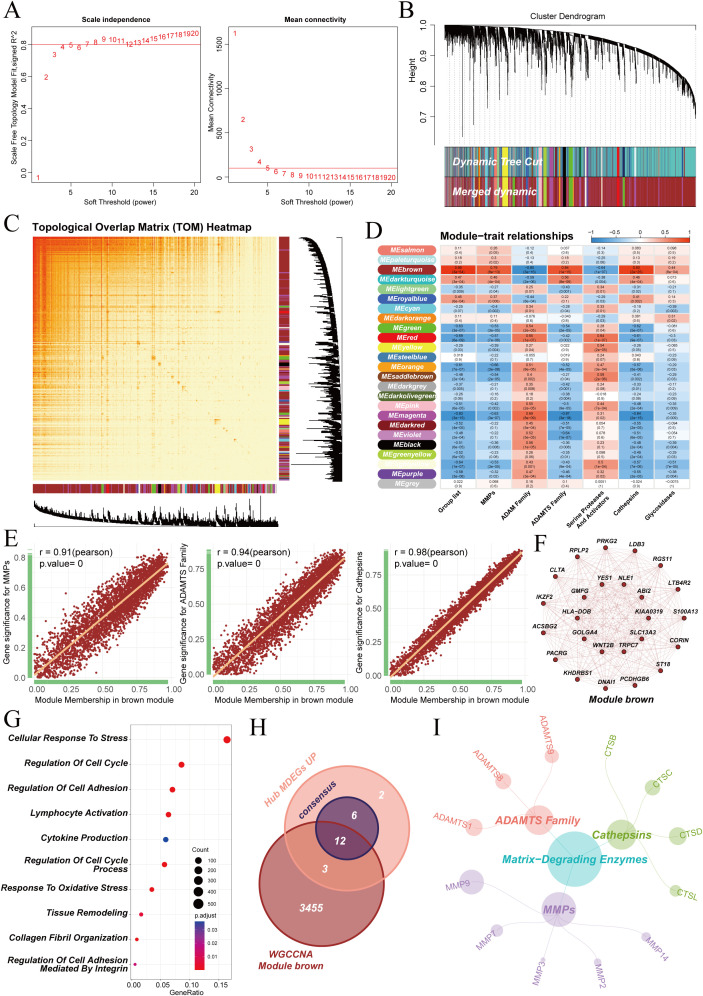
WGCNA Module Identification and Functional Analysis in IVDD. **(A)** Scale-free topology model fit index and mean connectivity for the selection of soft threshold power. **(B)** Cluster dendrogram of modules identified by hierarchical clustering. **(C)** Heatmap of the topological overlap matrix showing gene co-expression strength. **(D)** Module-trait relationships for key ECM degradation proteases and WGCNA modules. **(E)** Scatterplots showing the correlation between module membership in the brown module and gene significance for three key ECM degradation proteases. **(F)** Gene network diagram of genes in the brown module. **(G)** GO enrichment analysis of the brown module. **(H)** Venn diagram of the intersection between genes in the brown module, core ECM degradation genes and core protease genes. **(I)** Interaction network of twelve central genes and key proteases involved in ECM degradation.

WGCNA analysis was performed to systematically investigate the co-regulatory network of ECM degradation-related genes in IVDD using transcriptomic data. The scale-free topology was assessed, and a soft threshold power of 7 was selected (**Figure 3A**). Hierarchical clustering of genes identified 33 initial modules. These modules were merged into 25 distinct gene modules following dynamic cutting (height = 0.3) (**Figure 3B**). A topological overlap matrix heatmap was generated to visualize gene co-expression strength (**Figure 3C**). Module-phenotype correlation analysis revealed a significant positive correlation between the brown module and the scores of three key ECM degradation proteases: MMPs, ADAMTS, and cathepsins (**Figure 3D**). As a result, the brown module was selected for further analysis. Within this module, gene significance (GS) was strongly correlated with module membership (MM) (**Figure 3E**). This finding indicates that the genes associated with ECM degradation phenotypes also serve as pivotal hub genes that maintain the internal connectivity of the module.

Gene Ontology (GO) enrichment analysis was performed to explore the biological functions of the brown module. The results showed that genes in this module were significantly enriched in key pathways, including extracellular ECM remodeling, inflammation and immune activation, cellular stress, and programmed cell death (**Figure 3G**). This finding supports that the critical role of the brown module in IVDD. It coordinates four interrelated biological processes: ECM degradation, inflammatory response, cellular homeostasis imbalance, and apoptosis.

Genes from the brown module were intersected with core ECM degradation genes and the eighteen core protease genes to identify key genes. This refined set included 12 central genes (**Figure 3H**). The network diagram illustrates their relationships with the three types of core proteases, highlighting their involvement in the core regulatory network for ECM degradation in IVDD (**Figure 3I**).

### 3.4 Single-cell transcriptomics reveals ECM degradation dynamics in degenerative trajectories

**Figure 4:**
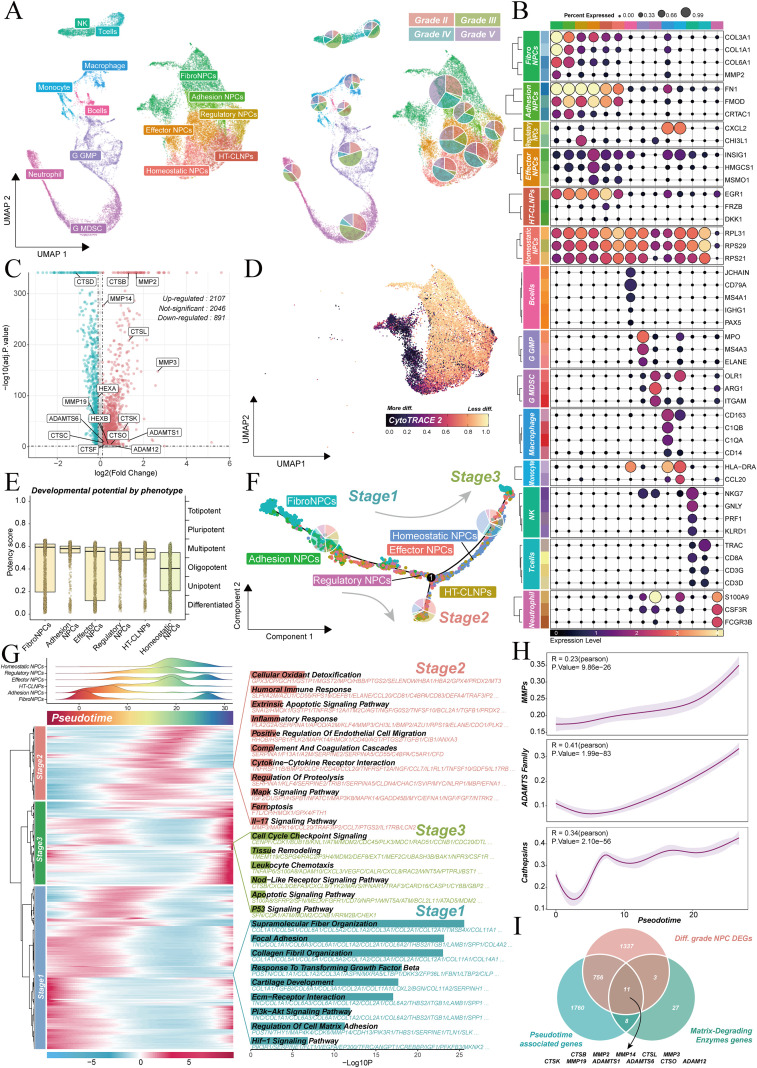
Dynamics of ECM degradation in intervertebral disc degeneration. **(A)** UMAP projection of human degenerated nucleus pulposus cells, classified into 14 subgroups including various NP cell types and immune cells (left). The proportions of each cell type across Pfirrmann grades I-V are shown (right). **(B)** Gene expression patterns for key cell types, identified by marker genes. **(C)** Volcano plot illustrating DEGs between NPCs from mild and severe degeneration. **(D)** CytoTRACE2 analysis revealing the differentiation trajectory from fibroNPCs to homeostatic NPCs. **(E)** Developmental potential scores of various phenotypes, categorized by differentiation stages. **(F)** Pseudotime trajectory analysis identifying three distinct stages of NP cell evolution during degeneration. **(G)** Heatmap depicting pseudotime-related gene expression dynamics across the three stages. **(H)** Correlation between pseudotime progression and ECM degradation, showing a significant increase in ECM degradation score. **(I)** Venn diagram highlighting 11 core genes involved in ECM degradation, identified from the intersection of genes related to degeneration severity, ECM degradation, and pseudotime.

Single-cell RNA sequencing data of human degenerated nucleus pulposus tissues (GSE165722) were analyzed to examine the processes of intervertebral disc degeneration (IVDD) at a cellular resolution. UMAP dimensionality reduction was applied, classifying cells into 14 subgroups (**Figures 4A**, left). These subgroups included various NP cell types, such as fibroNPCs, regulatory NPCs, effector NPCs, HT-CLNPS, and homeostatic NPCs, as well as immune cells, including macrophages, T cells, and neutrophils. **Figure 4A** (right) displays the proportions of I, II, III, IV, and V Pfirrmann grades for each cell type. Cell types were identified based on known marker genes (**Figure 4B**). Differential analysis was conducted between NPCs from mild (grades II-III) and severe (grades IV-V) degeneration to focus on cell populations most closely related to degeneration severity. This analysis revealed 2,107 upregulated genes (**Figure 4C**).

The CytoTRACE2 algorithm was used to infer differentiation potential, exploring cellular evolutionary trajectories during degeneration. The results suggested a potential differentiation trajectory from fibroNPCs to homeostatic NPCs (**Figures 4D, E**). Additionally, a pseudotime trajectory constructed using Monocle2 revealed the dynamic evolution of NP cells during degeneration (**Figure 4F**). The expression patterns of 2,535 genes highly correlated with pseudotime were used to divide the process into three distinct stages (**Figure 4G**). The first stage, the compensatory phase, is characterized by hypoxic adaptation mediated by HIF-1α signaling and ECM synthesis. The second stage, the decompensatory phase, is marked by the activation of inflammation, apoptosis, ECM degradation, and oxidative stress pathways, signaling the transition to irreversible tissue damage. The third stage, the exhaustion phase, is defined by cell apoptosis, immune dysregulation, and tissue fibrosis collapse, leading to the loss of disc function. Notably, the core ECM degradation score significantly increased as pseudotime progressed (**Figure 4H**), indicating that NPC cells enhance their ECM degradation capacity along the differentiation trajectory. This finding provides a theoretical basis for intervening in ECM degradation to delay the degeneration process.

Genes associated with degeneration severity, known ECM degradation genes, ECM degradation–related protease genes and pseudotime-related genes were intersected, leading to the successful identification of 11 core genes at the single-cell level (**Figure 4I**). These genes dynamically participate in ECM degradation during different stages of degeneration.

### 3.5 Integration of multiple algorithms for key gene selection and construction of an ECM degradation scoring model

**Figure 5:**
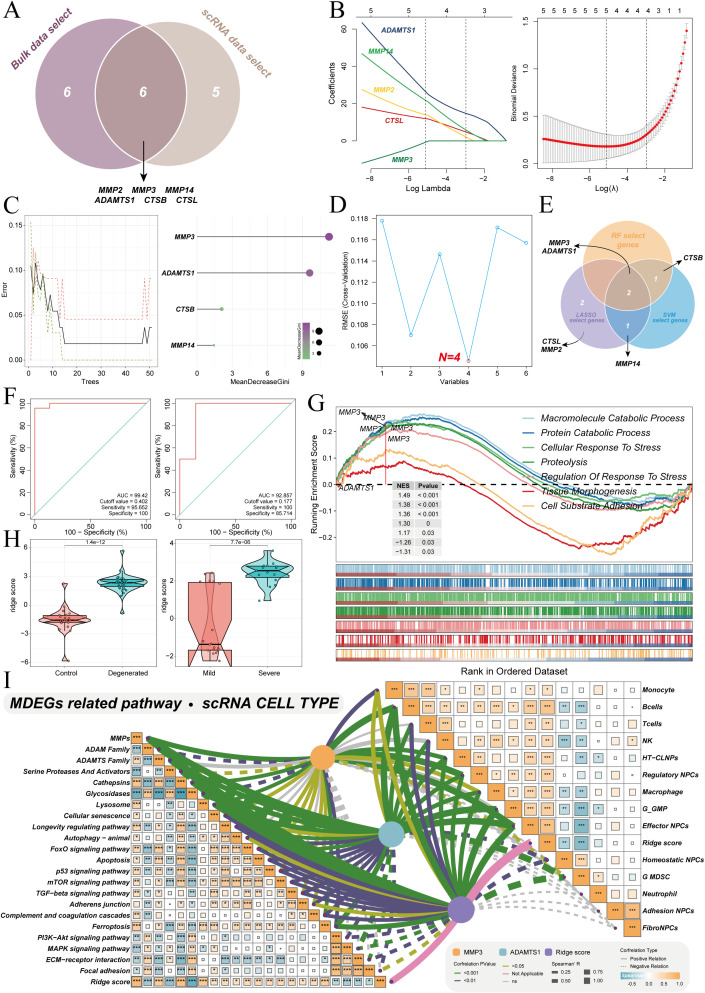
Integration of machine learning models for key ECM degradation genes. **(A)** Venn diagram illustrating the overlap of key genes identified from bulk and single-cell RNA sequencing data. **(B–D)** Gene selection via LASSO regression, Random Forest, and Support Vector Machine models. **(E)** Venn diagram showing intersection of genes selected by LASSO, Random Forest, and SVM algorithms. **(F)** ROC curve for the ECM degradation scoring model (MDEGs) in both training and validation sets. **(G)** Gene Set Enrichment Analysis (GSEA) highlighting enriched pathways in high versus low MDEGs groups. **(H)** Boxplots showing MDEGs scores across different degeneration grades. **(I)** Correlation matrix linking MDEGs scores, MMP3, ADAMTS1 expression, KEGG pathways and immune cell abundance. *p < 0.05, **p < 0.01, ***p < 0.001, ****p < 0.0001.

The core genes involved in ECM degradation during IVDD were identified by integrating previous analysis results. The 12 central genes from bulk transcriptomic data were intersected with the 11 core genes from single-cell data, leading to the identification of 6 key genes that were consistently found across multiple levels (**Figure 5A**). Three machine learning algorithms were applied to further refine this gene set. LASSO regression, using tenfold cross-validation, identified five feature genes (**Figure 5B**). Random forest, based on importance scores greater than 2, selected three core genes (**Figure 5C**). The SVM identified four specific genes (**Figure 5D**). The intersection of the results from all three algorithms revealed that MMP3 and ADAMTS1 were the key genes driving ECM degradation in IVDD (**Figure 5E**). A ridge regression model was constructed based on these two key genes to quantify individual ECM degradation risk (MDEG score or MDEGs). The samples were randomly divided into 70% training and 30% validation sets. ROC curve analysis showed that the scoring model exhibited excellent diagnostic performance in both the training and validation sets (**Figure 5F**), indicating its reliability in distinguishing degenerated from non-degenerated samples. The optimal cutoff, determined by the ROC curve, was used to divide the samples into high and low ECM degradation score groups. GSEA analysis revealed pathway differences between the two groups. The high-score group was significantly enriched in seven core pathways associated with MMP3 and ADAMTS1, focusing primarily on macromolecule catabolism and cellular stress processes (**Figure 5G**).

These findings suggest that these pathways may be key initiators of chronic inflammation and abnormal ECM remodeling in the later stages of IVDD. Consistent with this, the MDEGs score was significantly higher in the degeneration group compared to the non-degeneration group, and it was also notably higher in the severe degeneration group (grades IV-V) compared to the mild degeneration group (grades I-III) (**Figure 5H**). Further correlation analysis confirmed that the MDEGs score was significantly positively correlated with the activity of the aforementioned ECM degradation-related KEGG pathways (**Figure 5I**). Additionally, the expression of MMP3 and ADAMTS1, along with the MDEGs score, was positively correlated with the abundance of several pro-inflammatory immune cells and degeneration-related NPCs, such as regulatory NPCs, effector NPCs, and HT-CLNPS.

### 3.6 Validation of key genes at the single-cell level and uncovering transcriptional regulatory networks

**Figure 6:**
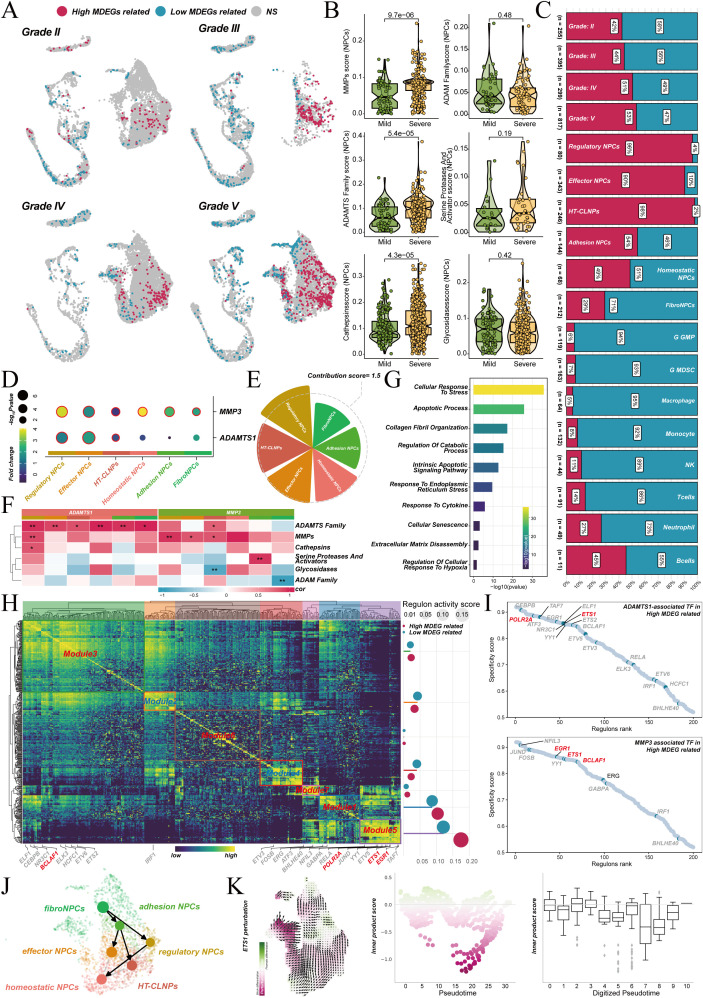
Validation of ECM degradation in NPCs and transcriptional networks in IVDD. **(A)** Single-cell analysis identifying cell subpopulations with high/low MDEG scores in different grades of degeneration. **(B)** Boxplots showing differential activity of ECM degradation pathways across mild and severe degeneration groups. **(C)** Bar chart illustrating the abundance of high/low MDEG scoring cells in different Pfirrmann grade and cells. **(D)** Heatmap showing expression of MMP3 and ADAMTS1 in different NPCs. **(E)** Contribution scores for different NPCs for IVDD. **(F)** Heatmap depicting gene expression correlation between MMP3, ADAMTS1, and ECM degradation proteases. **(G)** GO enrichment analysis highlighting key pathways involved in ECM degradation. **(H)** RAS of transcription factors in high MDEG-related cells, grouped into seven modules. **(I)** Ranking of transcription factors regulating MMP3 and ADAMTS1. **(J)** PAGA trajectory analysis of degenation related NPCs. **(K)** Transcription factor perturbation experiments showing the disruption of NPC fate transition upon ETS1 knockout. *p < 0.05,**p < 0.01, ***p < 0.001, ****p < 0.0001.

The functional significance of MMP3 and ADAMTS1 was validated at the single-cell level. The SCISSOR algorithm was used to identify cell subpopulations correlated with high MDEG score in the single-cell dataset (**Figure 6A**). The analysis showed that in NPC cells from the severe degeneration group (grades IV-V), the activity of MMPs, ADAMTS, and cathepsins pathways was significantly enhanced. In contrast, no significant differences were observed in the activity of other proteases (**Figure 6B**). Cells associated with high MDEG score were most abundant in severe degeneration samples. These cells were primarily concentrated in three NPC subtypes: regulatory NPCs, effector NPCs, and HT-CLNPS (**Figure 6C**). These findings suggest that these cell types are key drivers of ECM degradation. Further analysis showed that MMP3 and ADAMTS1 were significantly upregulated in the high-scoring cells identified by SCISSOR. These cells were primarily found in the three NPC subtypes mentioned earlier (**Figure 6D**). The cells also had the highest contribution scores to the severity of IVDD (**Figure 6E**). The expression of MMP3 and ADAMTS1 positively correlated with scores of various ECM degradation proteases (**Figure 6F**). Gene Ontology enrichment analysis revealed that gene expression changes in high MDEGs related NPC cells indicated a synergistic pathological network. This network centered on extracellular ECM degradation and involved collagen breakdown, abnormal activation of catabolic processes, cellular senescence, intrinsic apoptosis, and inflammatory responses (**Figure 6G**).

The Regulon Activity Scores (RAS) were calculated for each Regulon to assess differences in transcription factor activation across MDEGs scoring cell groups. These scores were clustered into seven distinct modules (M1-M7). Transcription factors regulating high MDEGs related cells were predominantly enriched in modules M1, M3, M5, M6, and M7 (**Figure 6H**). Transcription factors that regulated both MMP3 and ADAMTS1 were mainly concentrated in modules M1, M3, and M5. ETS1 was identified as the core transcription factor regulating both of these key genes (**Figure 6I**). PAGA trajectory analysis (**Figure 6J**) and subsequent transcription factor perturbation experiments confirmed that the knockout of ETS1 significantly disrupted the fate transition of degeneration associated NPC cells. These cells included regulatory NPCs, effector NPCs, and HT-CLNPS along the pseudotime trajectory (**Figure 6K**). These findings indicate that ETS1 plays a critical role in the transcriptional regulatory network of IVDD.

### 3.7 The role of cell communication and metabolic reprogramming in ECM degradation

**Figure 7:**
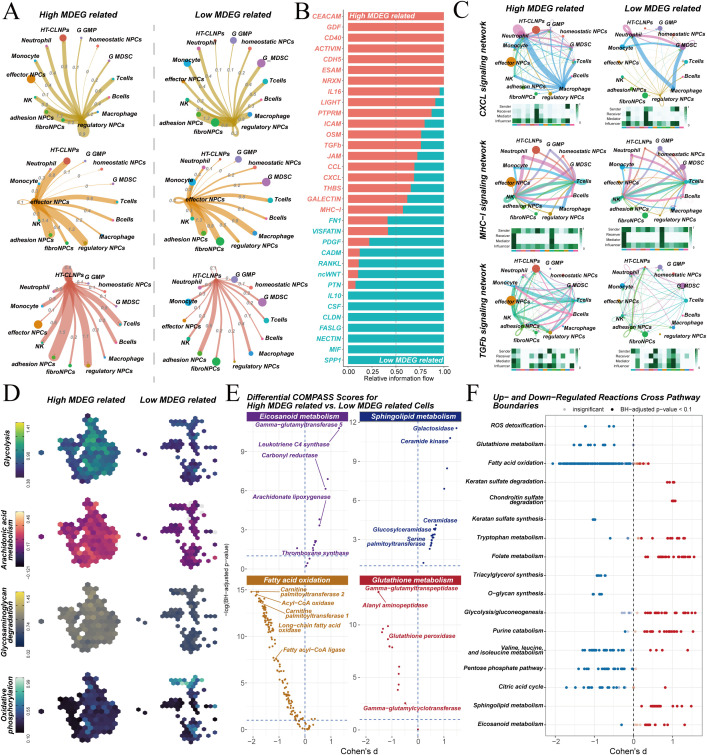
ECM degradation and metabolic reprogramming in MDEG-related cells. **(A)** Cell communication network analysis of high and low MDEG-related cells. **(B)** Differential communication pathways between high/low MDEGs related cells. **(C)** Signaling network analysis of CXCL, MHC-I, and TGF-β pathways in high/low MDEGs related cells. **(D)** Metabolic pathway analysis showing enhanced glycolysis and arachidonic acid metabolism in high/low MDEGs related cells. **(E)** Differential COMPASS scores for eicosanoid, sphingolipid, fatty acid oxidation and glutathione metabolism in high/low MDEGs related cells. **(F)** Cross-pathway analysis of upregulated and downregulated reactions across various metabolic pathways.

The microenvironmental characteristics of cells with high/low MDEGs were further explored. The interactions between MDEGs-related cells, identified by SCISSOR, were analyzed. Differences in cell communication associated with MDEGs scores were also examined. Regulatory NPCs, effector NPCs, and HT-CLNPS cells contained a higher proportion of MDEGs-related cells. Therefore, communication differences involving these cell types were likely more significant. As expected, cells with high MDEGs scores exhibited higher levels of cell communication (**Figure 7A**). Analysis of differential communication signals revealed that signaling pathways related to ECM remodeling, inflammatory responses, and cellular stress were significantly upregulated in the high-scoring group (**Figure 7B**). Specifically, these cells showed augmented chemotactic signaling (CXCL pathway), suggesting they actively recruit immune cells to amplify local inflammation. Activation of MHC-I signaling further implied potential antigen-presenting functions, which may drive autoimmune-like responses in IVDD. Moreover, excessive TGF-β1 signaling could promote fibrotic phenotype transformation, leading to metalloproteinase expression, collagen degradation, and proteoglycan loss, collectively disrupting ECM homeostasis (**Figure 7C**).

Distinct gene expression and communication patterns were exhibited by different NPC subtypes identified by SCISSOR. Metabolic changes in these cells were next assessed. Two algorithms, scMetabolism and Compass, were applied to evaluate their metabolic characteristics. scMetabolism analysis showed significantly enhanced glycolysis, arachidonic acid metabolism, and glycosaminoglycan degradation, alongside reduced oxidative phosphorylation (**Figure 7D**), indicating a shift toward a catabolic energy metabolism that supports their secretory and degradative functions. Compass analysis confirmed upregulation of eicosanoid metabolism (linked to inflammation) and sphingolipid metabolism (associated with apoptosis), whereas fatty acid oxidation and glutathione metabolism (essential for mitochondrial function and antioxidant defense) were downregulated (**Figure 7E**). These metabolic reprogramming events together contribute to a vicious cycle of energy metabolism imbalance, increased oxidative stress, and enhanced catabolism (**Figure 7F**). This cycle drives the pathological progression of IVDD.

These findings demonstrate that high MDEGs NPCs not only drive ECM degradation through MMP3/ADAMTS1 expression but also actively shape a pro-inflammatory microenvironment via chemokine signaling and undergo metabolic reprogramming to sustain their catabolic and inflammatory functions. This integrated analysis positions these cells as central hubs linking ECM degradation, immune dysregulation, and metabolic stress in IVDD, and highlights chemokine pathways (e.g., CXCL receptors) and metabolic pathways (e.g., those involved in arachidonic acid and sphingolipid metabolism) as potential therapeutic targets for disrupting this pathogenic network.

### 3.8 Drug target prediction and molecular docking validation

**Figure 8:**
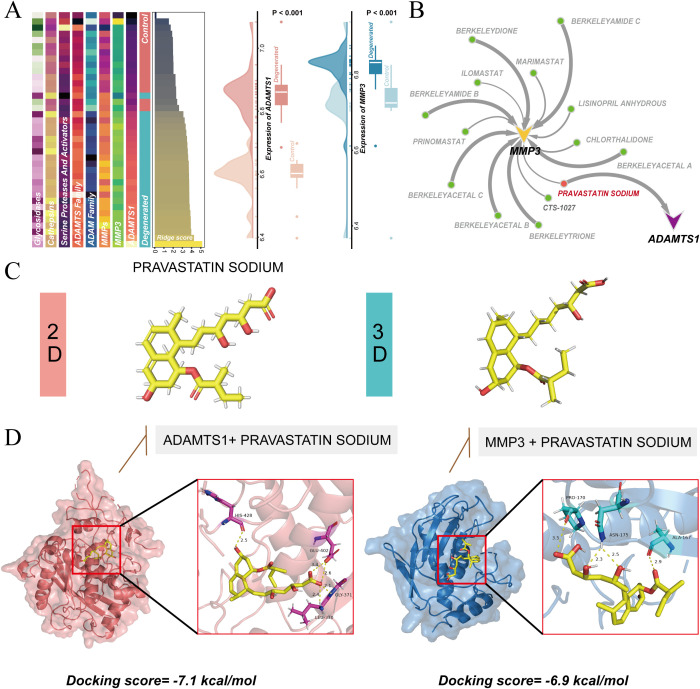
Drug screening and binding affinity analysis. **(A)** Expression changes of proteases and key genes as ridge score increases (Left panel). Expression analysis of MMP3 and ADAMTS1 in degenerated versus control samples (Right panel). **(B)** Drug-target interaction network highlighting pravastatin sodium interaction with ADAMTS1 and MMP3. **(C)** 2D and 3D chemical structures of pravastatin sodium. **(D)** Molecular docking results for pravastatin sodium with ADAMTS1 and MMP3.

The potential of MMP3 and ADAMTS1 as therapeutic targets was further explored. Samples were ranked according to MDEGs scores. A coordinated change in ECM degradation types was observed as the MDEGs score increased. MMP3 and ADAMTS1 showed significantly higher expression in the degeneration group compared to the control group (**Figure 8A**). This finding supports their reliability as key intervention targets.

The DsigDB database was used to screen for drug-gene interactions. Pravastatin sodium was identified as a small molecule interacting with both ADAMTS1 and MMP3 (**Figure 8B**). The 2D and 3D chemical structures of this drug are shown in **Figure 8C**. Molecular docking analysis was performed to validate the binding feasibility of pravastatin sodium. The results indicated that the binding energies of pravastatin sodium with ADAMTS1 and MMP3 were both below -5 kcal/mol (**Figure 8D**). These findings suggest strong and stable binding between pravastatin sodium and the two target proteins.

In summary, the dynamic expression profiles of MMP3 and ADAMTS1 provide new key targets for the treatment of IVDD. The high-affinity binding characteristics of pravastatin sodium offer a solid theoretical foundation for its future translational application and research.

### 3.9 *In vitro* and *in vivo* validation of pravastatin in alleviating IVDD

**Figure 9:**
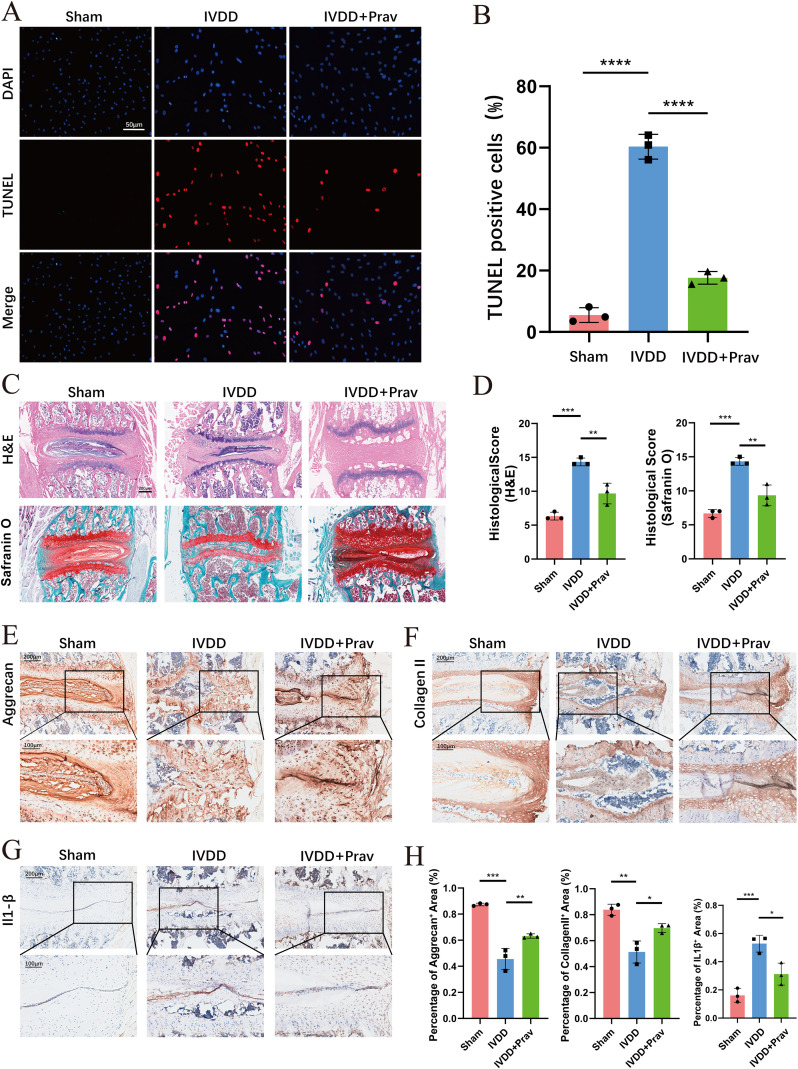
Pravastatin alleviates IVDD progression *in vitro* and *in vivo*. **(A)** Representative TUNEL staining showing apoptotic cells in NPC cultures with hydrogen peroxide-induced degeneration. **(B)** Quantification of TUNEL-positive cells. **(C)** H&E and Safranin O staining of mouse intervertebral discs. **(D)** Histological scores of H&E and Safranin O stained discs in three groups. **(E)** Immunohistochemistry of aggrecan expression. **(F)** Immunohistochemistry of collagen II expression. **(G)** Immunohistochemistry of IL-1β expression. **(H)** Quantification of aggrecan, collagen II, and IL-1β positive areas. Data represent mean ± SEM; *p < 0.05, **p < 0.01, ***p < 0.001, ****p < 0.0001.

The therapeutic potential of pravastatin sodium in IVDD was assessed through *in vitro* and *in vivo* models. An *in vitro* degeneration model was established by using hydrogen peroxide to induce NPC cell degeneration. The experiment included three groups: control, degeneration model, and pravastatin sodium treatment. TUNEL apoptosis assays were performed to evaluate cell death. The results showed a significant reduction in cell apoptosis in the pravastatin sodium treatment group compared to the degeneration model group (**Figures 9A, B**). This finding indicates that pravastatin sodium effectively counteracts cell death induced by the degenerative environment.

At the animal level, a mouse IVDD model was established. Treatment was evaluated through histological staining. Hematoxylin and eosin (HE) staining, along with Safranin O staining, showed that pravastatin sodium treatment significantly alleviated structural damage and loss in the nucleus pulposus tissue. These findings indicate that pravastatin sodium better maintains the normal morphology of the intervertebral disc (**Figures 9C, D**). Imunohistochemical staining revealed that pravastatin sodium treatment led to partial restoration of key ECM components, such as type II collagen and aggrecan, in the degenerated disc tissue. In contrast, the expression of the pro-inflammatory cytokine IL-1β was significantly suppressed (**Figures 9E–H**).

These findings from both cell and animal models consistently demonstrate that pravastatin sodium can delay the progression of IVDD. This is achieved by inhibiting cell apoptosis, reducing inflammation, and reducing ECM loss, thereby offering protective effects at multiple levels.

## 4 Discussion

IVDD is a leading cause of chronic low back pain and disability globally (39). Beyond the well-established paradigm of mechanical failure and ECM catabolism, our study underscores IVDD as a condition fueled by a dysregulated immune-metabolic microenvironment. We demonstrate that the key proteolytic drivers of ECM breakdown, MMP3 and ADAMTS1, are not merely downstream effectors of degeneration but are integral components of a self-amplifying pro-inflammatory circuit (6, 40). Notably, ECM integrity loss in the nucleus pulposus is linked to reduced disc hydration and height, exacerbating IVDD progression (11). Therefore, interventions targeting these processes are highly significant for treating IVDD.

In this study, five independent transcriptomic datasets (GSE167199, GSE176205, GSE34095, GSE56081, and GSE70362) were integrated to construct a comprehensive molecular landscape of ECM-degrading proteases associated with IVDD. Machine learning analysis identified MMPs, ADAMTS, and cathepsins as key proteases involved in ECM degradation. WGCNA and unsupervised clustering techniques were used to further pinpoint MMP3 and ADAMTS1 as key genes driving ECM breakdown in IVDD. A Ridge regression model, developed based on these two genes, demonstrated strong performance in distinguishing between degenerated and non-degenerated groups. Molecular docking analysis revealed that pravastatin sodium may serve as a potential therapeutic candidate for IVDD, showing strong binding affinities for both MMP3 and ADAMTS1. This was confirmed through *in vitro* and *in vivo* experiments, which demonstrated pravastatin sodium’s protective effects on ECM integrity and its ability to modulate inflammatory responses in IVDD models. These findings highlight MMP3 and ADAMTS1 as crucial biomarkers and therapeutic targets for IVDD. They also offer new insights into the regulation of ECM degradation and potential strategies for disease intervention.

Key proteases identified in this research include MMPs, ADAMTS, and cathepsins which is consistent with recent research (6). Notably, although the involvement of MMP3 and ADAMTS1 in IVDD has been sporadically reported, these studies largely focused on individual genes without systematic comparison of their relative importance within the matrix-degrading enzyme repertoire (40–43). In this study, by integrating multi-source omics data (including bulk microarray, RNA-seq, and single-cell sequencing data) and multiple machine learning algorithms, we have, for the first time, identified MMP3 and ADAMTS1 as core regulatory genes from a comprehensive set of matrix-degrading genes, revealing their dominant role in IVDD progression. Matrix metalloproteinases (MMPs), particularly MMP-3 identified in our study, exhibit broad substrate specificity and are persistently upregulated by pro-inflammatory cytokines such as IL-1β and TNF-α, thereby contributing to ECM degradation in IVDD (12, 44). ADAMTS enzymes, including ADAMTS1, mainly target aggrecan, and their increased activity during IVDD promotes proteoglycan loss and disruption of ECM homeostasis (45–47). Cathepsins, especially cathepsin B and cathepsin K, participate in both intracellular and extracellular matrix degradation and are also associated with apoptosis and inflammatory responses (48–51).

In this study, various machine learning techniques have highlighted MMP-3 (also referred to as stromelysin-1) and ADAMTS1 as key genes. MMP-3 demonstrates broad substrate specificity, contributing to the degradation of various proteoglycans and other non-fibrillar ECM components (52). Investigations into human intervertebral disc tissues with degeneration have demonstrated a marked increase in MMP-3 expressionf IVD matrix components.•MMPs and ADAMTSs are expressed in (53). In clinical cohorts, elevated MMP-3 transcript levels within the disc correlate with higher Pfirrmann MRI grades, suggesting a strong association with radiological degeneration. Additionally, Mendelian randomization studies conducted in European populations imply a potential causal relationship between increased plasma MMP-3 levels and the susceptibility to intervertebral disc degeneration, thereby supporting the “systemic MMP-3 burden–disc degeneration” hypothesis.

Notably, MMP3 and ADAMTS1 expression showed significant positive correlations with the infiltration abundance of multiple pro-inflammatory immune cells, indicating a close interplay between ECM degradation and immune microenvironment remodeling. ECM degradation fragments can act as damage-associated molecular patterns (DAMPs), activating innate immune cells via TLR receptors and promoting the release of pro-inflammatory cytokines such as IL-1β and TNF-α; these cytokines in turn further upregulate MMP3 and ADAMTS1 expression, forming a “degradation-inflammation-redegradation” positive feedback loop. Both IL-1β and TNF-α have been shown to consistently upregulate MMP-3 expression, suggesting that targeting MMP-3 and its upstream inflammatory signaling pathways may provide a viable approach to slowing ECM degradation and preserving disc structural integrity. For ADAMTS1, in a chronic LPS-induced inflammatory mouse model, p38-mediated phosphorylation of Runx2 promotes the recruitment of p300/NCOA3, enhancing ADAMTS1 expression and accelerating ECM degradation (54).

Single-cell analysis further revealed that high MDEGs-scoring NPC subpopulations not only highly expressed MMP3 and ADAMTS1 but also exhibited enhanced chemokine signaling and MHC-I signaling, suggesting these cells may actively participate in immune cell recruitment and antigen presentation. Concurrently, their metabolic shift toward glycolysis and arachidonic acid metabolism supports the energetic demands of their pro-inflammatory and catabolic activities, while increased sphingolipid metabolism links to apoptosis susceptibility. This integrated view positions MDEGs-high cells as central hubs linking ECM degradation, immune dysregulation, and metabolic stress, and highlights chemokine receptors and metabolic enzymes as novel therapeutic targets. However, it should be noted that these immune-related conclusions are primarily based on transcriptomic inference and require validation through flow cytometry, immunofluorescence co-localization, and functional blockade experiments in future studies.

Pravastatin is a widely studied statin that reduces LDL-C levels by inhibiting HMG-CoA reductase (55). Beyond its lipid-lowering effects, statins have been extensively investigated for their pleiotropic properties (56), including anti-inflammatory (57, 58), antioxidant (59), endothelial function improvement (60), and regulation of autophagy (61). Numerous observational studies and systematic reviews suggest associations between statin use and degenerative diseases such as dementia (62), Parkinson’s disease (63), and multiple sclerosis (64), as well as musculoskeletal disorders like osteoarthritis (65) and IVDD (66). Large-scale observational studies indicate that individuals with hypercholesterolemia who have a higher cumulative statin dose may experience a reduced risk of spinal degenerative joint disease (67). but the available evidence is largely based on clinical and indirect data (68, 69). In the present study, molecular docking indicated strong binding affinity of pravastatin for MMP3 and ADAMTS1. These in silico findings were supported by *in vitro* and *in vivo* experiments showing that pravastatin preserved ECM integrity, suppressed IL-1β expression, and reduced NPC apoptosis. Collectively, these results identify pravastatin as a potential repurposing candidate for targeting the MMP3/ADAMTS1-mediated degeneration–inflammation axis in IVDD.

The translational relevance of pravastatin nevertheless requires careful consideration. The administration route and dosage used here were selected on the basis of standard mouse-model practice and previously reported effective statin regimens (70–73). However, delivery of systemically administered agents to the nucleus pulposus remains a major challenge because the intervertebral disc relies primarily on diffusion through the endplate. Available evidence indicates that systemic compounds can reach articular cartilage and disc tissue, particularly under inflammatory conditions, when endplate microstructural disruption may increase permeability (74–76). Whether such delivery achieves sufficient and sustained intradiscal exposure for therapeutic efficacy remains uncertain.

Importantly, the anti-apoptotic effect of pravastatin is unlikely to be explained solely by predicted inhibition of MMP3 and ADAMTS1. This effect may instead reflect broader pleiotropic actions, including suppression of apoptosis through anti-inflammatory, antioxidant, or autophagy-related mechanisms. An indirect effect is also plausible, as preservation of ECM integrity may stabilize cell–matrix interactions, reduce anoikis, and interrupt the degeneration–inflammation–apoptosis cascade. Selective inhibition and genetic validation studies are needed to distinguish these mechanisms. Pharmacokinetic analysis, evaluation of intradiscal or other local delivery approaches, and long-term safety assessment will also be necessary to define the therapeutic potential of pravastatin in IVDD.

Despite the promising results, several limitations should be noted in this study. First, while the integration of multiple transcriptomic datasets provided a comprehensive molecular landscape, variations between the datasets, such as batch effects and differences in experimental conditions, could introduce biases that affect the robustness of the findings. Second, although pravastatin sodium showed strong binding affinities in molecular docking and protective effects in *in vitro* and *in vivo* experiments, the precise mechanisms underlying its action on MMP3 and ADAMTS1 require further clarification. Additionally, the potential off-target effects of pravastatin sodium and its long-term safety profile in the context of IVDD need to be investigated in more detail. Fourth, the sample size in the animal experiment (n=3 per group) is relatively small, which may limit the statistical power and generalizability of the findings. This exploratory study was designed primarily to assess the preliminary therapeutic trend of pravastatin in IVDD. Future studies with larger sample sizes (n ≥ 6 per group) are warranted to confirm the efficacy observed herein and to enable robust statistical comparisons, including subgroup analyses and long-term safety assessments. Future research should explore alternative therapeutic candidates and their interactions with the ECM to refine treatment strategies for IVDD.

In conclusion, This study presents a detailed molecular landscape of ECM-degrading proteases in the context of IVDD by integrating five independent transcriptomic datasets. Through machine learning and bioinformatic analyses, we identified MMP3 and ADAMTS1 as pivotal drivers of ECM degradation, whose expression is closely linked to the pro-inflammatory immune microenvironment. The development of a Ridge regression model based on these genes demonstrated robust discriminatory power between degenerated and non-degenerated disc groups. Additionally, molecular docking simulations suggested that pravastatin sodium could be a promising therapeutic agent for IVDD by targeting MMP3 and ADAMTS1, which was further validated in both *in vitro* and *in vivo* experiments. This research underscores the importance of MMP3 and ADAMTS1 as key biomarkers for IVDD and provides a foundation for future therapeutic strategies aimed at modulating ECM degradation and inflammation in IVDD.

## Data Availability

The original contributions presented in the study are included in the article/**Supplementary Material**. Further inquiries can be directed to the corresponding author.

## Supplementary material

Supplementary Table 1 Information on genes associated with proteases degrading the extracellular matrix.
