# Supplementary material for: Deciphering the regulatory mechanism and therapeutic potential of ECM degradation in intervertebral disc degeneration via multi-omics integration
Source: Front Immunol. 2026 Apr 22;17:1809762. doi: 10.3389/fimmu.2026.1809762 (PMC13143665; doi:10.3389/fimmu.2026.1809762)
Supplement: Supplementary Table 1 — Information on genes associated with proteases degrading the extracellular matrix. [file Table1.docx]

**Matrix Metalloproteinases (MMPs)** [1, 2]**:** MMP2, MMP3, MMP7, MMP9, MMP11, MMP13, MMP14, MMP15, MMP16, MMP19, MMP24, MMP25
**A Disintegrin and Metalloproteinase (ADAM) Family**[3]**:** ADAM8, ADAM9, ADAM12, ADAM15, ADAM17, ADAM19, ADAM22, ADAM28
**A Disintegrin and Metalloproteinase with Thrombospondin Motifs (ADAMTS) Family**[3]**:** ADAMTS1, ADAMTS2, ADAMTS6, ADAMTS8, ADAMTS9, ADAMTS12, ADAMTS13
**Serine Proteases and Activators**[4, 5],**:** PLAU, KLK11, KLK14, GZMB, GZMM, ST14
**Cathepsins (Cysteine Proteases)** [6]**:** CTSB, CTSC, CTSD, CTSF, CTSH, CTSK, CTSL, CTSO
**Glycosidases:** HYAL1[7], HYAL2[7], HPSE[8], GUSB[9], GLB1[10], HEXA[11], HEXB[11], IDS[12].

**Reference**

1. Wang X, Khalil RA (2018) Matrix Metalloproteinases, Vascular Remodeling, and Vascular Disease. Adv Pharmacol 81:241–330. https://doi.org/10.1016/bs.apha.2017.08.002

2. Liu Z-L, Chen H-H, Zheng L-L, et al (2023) Angiogenic signaling pathways and anti-angiogenic therapy for cancer. Signal Transduction Targeted Ther 8:198. https://doi.org/10.1038/s41392-023-01460-1

3. Zhong S, Khalil RA (2019) A Disintegrin and Metalloproteinase (ADAM) and ADAM with thrombospondin motifs (ADAMTS) family in vascular biology and disease. Biochem Pharmacol 164:188–204. https://doi.org/10.1016/j.bcp.2019.03.033

4. Wenta T, Nastaly P, Lipinska B, Manninen A (2024) Remodeling of the extracellular matrix by serine proteases as a prerequisite for cancer initiation and progression. Matrix Biol 134:197–219. https://doi.org/10.1016/j.matbio.2024.10.007

5. Solomonov I, Kollet O, Sagi I (2025) Extracellular matrix and proteolysis: mechanisms driving irreversible changes and shaping cell behavior. FEBS J. https://doi.org/10.1111/febs.70292

6. Fonović M, Turk B (2014) Cysteine cathepsins and extracellular matrix degradation. Biochim Biophys Acta, Gen Subj 1840:2560–2570. https://doi.org/10.1016/j.bbagen.2014.03.017

7. Kim J, Seki E (2023) Hyaluronan in liver fibrosis: basic mechanisms, clinical implications, and therapeutic targets. Hepatology Communications 7:e0083. https://doi.org/10.1097/HC9.0000000000000083

8. Tripathi CKM, Banga J, Mishra V (2012) Microbial heparin/heparan sulphate lyases: potential and applications. Appl Microbiol Biotechnol 94:307–321. https://doi.org/10.1007/s00253-012-3967-6

9. Grant CL, López-Valdez J, Marsden D, Ezgü F (2024) Mucopolysaccharidosis type VII (Sly syndrome) - What do we know? Mol Genet Metab 141:108145. https://doi.org/10.1016/j.ymgme.2024.108145

10. Caciotti A, Donati MA, Boneh A, et al (2005) Role of beta-galactosidase and elastin binding protein in lysosomal and nonlysosomal complexes of patients with GM1-gangliosidosis. Hum Mutat 25:285–292. https://doi.org/10.1002/humu.20147

11. Hepbildikler ST, Sandhoff R, Kolzer M, et al (2002) Physiological substrates for human lysosomal beta -hexosaminidase S. J Biol Chem 277:2562–2572. https://doi.org/10.1074/jbc.M105457200

12. Demydchuk M, Hill CH, Zhou A, et al (2017) Insights into Hunter syndrome from the structure of iduronate-2-sulfatase. Nat Commun 8:15786. https://doi.org/10.1038/ncomms15786
